# A Review of Non-Pharmacological Treatments for Pain Management in Newborn Infants

**DOI:** 10.3390/children5100130

**Published:** 2018-09-20

**Authors:** Avneet K. Mangat, Ju-Lee Oei, Kerry Chen, Im Quah-Smith, Georg M. Schmölzer

**Affiliations:** 1 Faculty of Science, University of Alberta, Edmonton, AB T6G 2R3, Canada; amangat@ualberta.ca; 2 Centre for the Studies of Asphyxia and Resuscitation, Neonatal Research Unit, Royal Alexandra Hospital, Edmonton, AB T6G 2R3, Canada; 3 School of Women’s and Children’s Health, University of New South Wales, Kensington, NSW 2031, Australia; j.oei@unsw.edu.au; 4 Faculty of Medicine, University of New South Wales, Kensington, NSW 2033, Australia; kerry.chen@unsw.edu.au; 5 School of Women’s and Children’s Health University of New South Wales, Kensington, NSW 2033, Australia; quahsmith@gmail.com; 6 Department of Pediatrics, University of Alberta, Edmonton, AB T6G 2R3, Canada

**Keywords:** infant, premature, pain, acupuncture, skin-to-skin contact, sucrose, massage, musical therapy, breastfeeding

## Abstract

Pain is a major problem in sick newborn infants, especially for those needing intensive care. Pharmacological pain relief is the most commonly used, but might be ineffective and has side effects, including long-term neurodevelopmental sequelae. The effectiveness and safety of alternative analgesic methods are ambiguous. The objective was to review the effectiveness and safety of non-pharmacological methods of pain relief in newborn infants and to identify those that are the most effective. PubMed and Google Scholar were searched using the terms: “infant”, “premature”, “pain”, “acupuncture”, “skin-to-skin contact”, “sucrose”, “massage”, “musical therapy” and ‘breastfeeding’. We included 24 studies assessing different methods of non-pharmacological analgesic techniques. Most resulted in some degree of analgesia but many were ineffective and some were even detrimental. Sucrose, for example, was often ineffective but was more effective than music therapy, massage, breast milk (for extremely premature infants) or non-invasive electrical stimulation acupuncture. There were also conflicting results for acupuncture, skin-to-skin care and musical therapy. Most non-pharmacological methods of analgesia provide a modicum of relief for preterm infants, but none are completely effective and there is no clearly superior method. Study is also required to assess potential long-term consequences of any of these methods.

## 1. Introduction

Newborn infants admitted to an Neonatal Intensive Care Unit (NICU) undergo an average of 134 painful procedures within the first two weeks [1,2]. Even more concerning, some infants might experience more than 3000 painful procedures during the entire course of their NICU stay [3]. These procedures are often necessary to ensure best care, such as heel pricks for blood sampling or endotracheal suctioning. Some of these procedures are also performed repeatedly on the same infant and have been shown to cause adverse physiological consequences, such as hypoxemia, bradycardia and hypertension [1].

Apart from acute discomfort, there is now growing evidence, that painful (and particularly, repetitive) procedures may have adverse consequences on long-term neurological development. Animal models demonstrate that painful events in early life can increase the number of glucocorticoid receptors in the hippocampus, which may affect future stress response [4]. Pain may not even need to be chronic or repetitive to elicit adverse future outcomes. For example, infants have increased stress behavior during routine immunizations at 4–6 months if circumcision was conducted at birth without analgesia [5]. This highlights the lifelong implications of pain management during this critical period in life [1,6].

Pharmacological methods are the most frequently used means to ameliorate or prevent pain in infants. However, many analgesics have adverse effects. For example, opioids can have troublesome adverse effects including somnolence, and respiratory depression making it unsuitable for use in spontaneously breathing, opioid-naïve patients [7].

Adjuvants to pharmacological analgesia are therefore needed. Non-pharmacological analgesic methods include acupuncture, non-nutritive sucking (NNS), breastfeeding (BF), sucrose/glucose solution, skin-to-skin care (SSC), swaddling, therapeutic massage, musical therapy (MT) and facilitated tucking (Table 1). These methods utilize environmental, behavioral, and pharmacological approaches by activating a “gate control mechanism” that prevents the pain sensation from traveling to the central nervous system [8]. While evidence exists for non-pharmacological analgesic methods, the strategies are not used universally. We therefore aimed to summarize the current evidence about the efficacy, safety, and feasibility of non-pharmacological interventions for pain management in newborn infants to determine if they could be considered an alternative to other methods of analgesia, including medications.

## 2. Methods

PubMed and Google Scholar were systematically searched include the following search-terms “infants”, “pain”, “acupuncture”, “skin-to-skin contact”, “sucrose”, “massage”, “musical therapy” and “breastfeeding” between 1965 and 2018 (Appendix A). List of references of identified articles were manually searched. Articles were included if they described non-pharmacological techniques in preterm or term infants and excluded if they compared pharmacological and non-pharmacological intervention or there was no behavioral measurement of pain (e.g., PIPP (Premature Infant Pain Profile), or NIPS (Neonatal Infant Pain Scale)). Only human studies were included and no language restrictions were applied.

## 3. Results

A total of 26 studies describing acupuncture (*n* = 3), skin to skin care (*n* = 3), non-nutritive sucking (*n* = 1), swaddling (*n* = 3), sucrose/glucose solution (*n* = 3), massage (*n* = 4), musical therapy (*n* = 5), breastfeeding (*n* = 3) and facilitated tucking (*n* = 1) were identified (Table 2). A total of 10 studies were done in preterm infants, 12 studies were in term infants and four studies were done in preterm and term infants. It was not possible to make a distinction between preterm and term infants since many studies did not separate their samples by gestation.

### 3.1. Environmental Control

Creating a more comforting environment with SSC, swaddling, therapeutic touch/massage, music therapy and comfort positioning for the infant can produce analgesic effects. Cholecystokinin, a neuropeptide associated with analgesia, is released when the infant is exposed to familiar smell of the mother [32]. Therefore, providing an infant with SSC with the mother can have an analgesic effect [32]. Swaddling, the practice of wrapping infants in blankets and can help simulate the environment of the womb which may translate to analgesia [33,34]. Massage can potentially saturate the senses and decrease the pain signals that are sent to the central nervous system [35]. Music uses distraction to activate the infant’s attention and thus distracts them from the pain and decreases their sensation of pain [9]. Holding the infant in a flexed position, which is known as facilitated tucking, can also have analgesic effects due to saturation of senses similar to massage therapy [35].

#### 3.1.1. Skin-to-Skin Care 

Overall, most studies reported decreased pain responses during SSC compared to placebo methods. Seo et al. reported 35% less pain and an 88% decrease in crying duration with SSC compared to controls during heel pricks [10]. Similarly, Freire et al. compared SSC with oral glucose solution or placebo for pain relief of 95 preterm infants during heel prick. The infants randomized to SSC had significantly lower heart rates (mean ± standard deviation (SD), 5 ± 4 vs. control 11 ± 7 vs. oral glucose 10 ± 6 beats per minute (bpm); *p* = 0.0001) and oxygen saturation variation (1.5 ± 1.7 vs. control 2.6 ± 1.5 vs. oral glucose 1.9 ± 1.5%; *p* = 0.0012) than those given glucose or to controls [11]. Olsson et al. reported similar pain scores between SSC and placebo during venepuncture of 10 preterm infants [12]. In summary, SSC might reduce pain in preterm and term infants.

#### 3.1.2. Swaddling

Efendi et al. randomized 30 preterm infants to either swaddling and pacifier or control during painful procedures and demonstrated a significantly lower heart rate and pain scores in infants receiving swaddling and a pacifier [13]. As the study combined NNS and swaddling, it is therefore impossible to differentiate which of these methods might have provided pain relief. Other studies have reported that swaddling alone decreases pain during heel prick [14,15]. Erkut et al. reported a 50% pain reduction, a 30% decrease in duration of crying time, and a significantly increased oxygen saturation (mean ± SD, 97 ± 2 vs. control: 95 ± 2%, *p* = 0.006) in the swaddled group after a heel prick procedure in 74 term infants [14]. Ho et al. randomized 54 premature infants to swaddling or control and reported lower pain scores (7 ± 3 vs. control: 145 ± 3, *p* < 0.001), lower heart rate (162 ± 10 vs. control: 182 ± 17 bpm, *p* < 0.001), and higher oxygen saturation (96 ± 4 vs. control 87 ± 7%, *p* < 0.001) after heel prick [15]. These studies suggest that swaddling alone or combining with a pacifier has the potential to decrease pain in preterm and term infants.

#### 3.1.3. Facilitated Tucking

Axelin et al. randomized 20 preterm infants to either control or facilitated tucking (flexed position by their parents) [16]. Overall, facilitated tucking reduced pain by 40% (*p* < 0.001) and crying time was significantly shorter 5 s vs. 17 s, *p* = 0.024 when compared to no tucking [16].

#### 3.1.4. Therapeutic Touch/Massage

Two observational studies reported that massage therapy significantly reduced mean crying time (4.4 ± 1.8 vs. control: 5.3 ± 1.7 h/day, *p* < 0.001) in infants with infant colic [17] and decreased NIPS scores (3.9 vs. control: 4.8, *p* = 0.002) in term infants [34]. Two randomized trials reported that an upper limb massage significantly decreased pain responses during venipuncture in preterm and term infants [18,19]. Chik et al. randomized 80 infants and found significantly lower pain scores between the massage and control group (−6.0, *p* < 0.001) [18]. Similarly, Jain et al. found a 60% decrease in pain and a significant decrease in heart rate (mean ± SD: 149 ± 14 vs. control: 159 ± 13 bpm, *p* = 0.03) after venipuncture of 23 infants [19]. These studies suggest that a gentle massage prior a heel prick is safe and can decrease pain.

#### 3.1.5. Musical Therapy

Using MT, a case study with five infants in a cardiac intensive care unit showed a decreased average heart rate in 4/5 infants in 66% of the sessions [36]. In addition, respiratory rate and blood pressure were also decreased, while oxygen saturation increased in some of the infants [36]. Furthermore, Olischar et al. reported more mature sleep-wake cycles in newborn infants > 32 weeks’ gestation exposed to music when compared to controls suggesting a calming effect on quiet sleep [37]. These data suggest that MT has a stabilizing effect on physiological parameters and sleep, which could be translate to a decreased pain response. However, currently available evidence has conflicting results.

Shabani et al. randomized 20 preterm infants to MT or control during venous blood sampling and reported a significant decrease in heart rate (mean ± SD: 148 ± 4 vs. control 163 ± 4 bpm, *p* = 0.005) and an 80% decrease in the infants’ mean facial pain expressions with MT [9]. Zhu et al. randomized 250 term infants to either MT, MT + BF, BF, or control and observed that the BF group had a 50% decrease in pain and a 70% decrease in duration of crying time [20]. In addition, no difference between BF or BF + MT was observed, suggesting that MT was ineffective for pain relief [20]. Shah et al. randomized 35 infants to MT, sucrose, or MT + sucrose using a cross-over design [21]. Overall, median interquartile range pain scores were significantly lower in the MT + sucrose group (3, 0–4) compared to MT (6, 3–11) or sucrose (5, 3–10) alone [21]. In addition, pain scores were similar between the MT and sucrose groups [21]. These data suggest that a combination of MT + sucrose provides improved pain relief compared to sucrose or music alone.

### 3.2. Feeding Methods

Breastfeeding and NNS are believed to support analgesia through the release of cholecystokinin (neuropeptide) [32,38]. With BF, cholecystokinin is believed to be released due to familiar odors of the mother [32]. Non-nutritive sucking has been associated with the activation of sensory nerves that can lead to the release of cholecystokinin which can then interact with opioids and produce analgesia [1].

#### 3.2.1. Breastfeeding

Zurita-Cruz et al. randomized 144 infants to either BF, milk substitute, or no analgesia (control group) during vaccination and reported that infants who received BF had reduced pain and 50% reduction in median crying time compared with milk substitute or controls [22]. There were no significant differences in any of the parameters between the milk substitute and control groups suggesting that milk substitute was ineffective at decreasing pain [22]. Similarly, Erkul et al. randomized 100 infants to either BF or control prior vaccination and observed lower pain scores (mean ± SD: 1.9  ± 2.2 vs. 6.8  ± 0.7, *p* < 0.05), lower duration of crying (mean ± SD: 20.5 ± 16.2 vs. 45.1 ± 14.5 s, *p* = 0.005), lower heart rate (mean ± SD: 164  ± 17 vs. 172  ± 15 bpm, *p* < 0.05), and higher mean oxygen saturation (mean ± SD: 98  ± 3 vs. 94  ± 7%, *p* < 0.05) [23]. One may assume that there is a difference between the analgesic effects of BF compared to oral administering breast milk, due to BF having the additive analgesic effects from other factors such as parental presence and SSC. However, a randomized controlled trial of 71 preterm neonates reported a surprising distinction between BF and bottle feeding [24]. Bottle feeding significantly decreased the mean COMFORTneo pain score compared to breast feeding (BF: 19.0 vs. bottle-fed: 16.3, *p* = 0.03) [24]. The study also compared the analgesic effects of breast milk to sucrose and found no significant differences in PIPP scores eluding that both were just as effective [24]. It is also noteworthy that, in premature infants, even the smell of breast milk had a 50% reduction in pain scores during venipuncture and a decrease in percent of duration of crying (0.17 ± 0.6 vs. 9.7 ± 17.3 s, *p* = 0.04) after venipuncture [25]. These results are important in situations where breast feeding is not feasible in the NICU (e.g., mother not present) since the smell of breast milk has the potential to decrease pain.

#### 3.2.2. Non-Nutritive Sucking

A case-control study compared infants who sucked on an adult’s little finger (*n* = 20) with BF (*n* = 20) and without any analgesia (*n* = 23, control group) during venipuncture [39]. Overall outcomes with BF and NNS were similar (35 vs. 24%, *p* > 0.05) suggesting a similar efficacy in analgesia [39]. While NNS has some effect on pain relief further studies are needed to examine different approaches of NNS (e.g., use of finger or pacifier) or combination with glucose.

### 3.3. Other Interventions

#### 3.3.1. Acupuncture

Body acupuncture and pain management is well known with the spinal pain pathways being recruited for attenuation of pain signaling in needle acupuncture. Manual acupuncture activates different afferent fibers (Aβ, Aδ, and C) and these signals ascend mainly through the spinal ventrolateral funiculus to the brain [26]. Many brain nuclei compose the network involved in processing acupuncture analgesia such as locus coeruleus and arcuate nucleus [26]. However, more recent research reveals the bigger impact of acupuncture on the individual. Now considered a complex sensory stimulation, acupuncture effects also include autonomic re-regulation and regulatory changes in functional connectivity centrally—mitigating the effects of physical and emotional trauma in the individual [40,41]. The non-invasive approach (no needling modalities of acupuncture such as low-level laser and magnet application), is more autonomically driven to gain direct access to central pain control centers [40]. Neuroimaging studies have confirmed the re-regulatory capacity of acupuncture centrally [42].

Several studies examined the effects of non-invasive acupuncture on neonatal pain [27,28,43]. Chen et al. randomized 30 term infants to either auricular non-invasive magnetic acupuncture or placebo to decrease infant pain during heel pricks [27]. The study reported a 30% reduction in pain in infants receiving magnetic acupuncture [27]. Abbasoglu et al. reported a 45% reduction in mean duration of crying with acupressure compared to control infants during heel pricks in 32 preterm infants, but no significant differences in pain score between groups [28]. Differences in these studies could have been due to the use of varying techniques (e.g., acupuncture or acupressure) or different acupuncture points. Future studies should distinguish between the optimal points and duration of treatment (e.g., duration of placement of acupuncture) and to elucidate the long-term implications of different methods of acupuncture.

#### 3.3.2. Sucrose/Glucose Solutions

Oral sucrose solution is most commonly used as non-pharmacological interventions for pain management in newborn infants. Sucrose may exert its analgesic effects through endogenous opioid pathways or via an increase in dopamine and acetylcholine [1,44]. However, the evidence for pain relief is conflicting. Lima et al. reported a 40% reduction in pain scores and a 70% reduction in crying time with oral glucose compared to NNS in 78 healthy newborns during immunization [29]. Gouin et al. randomized 1–3 months old children undergoing a venipuncture to either sucrose or placebo and found similar pain scores (mean ± SD: sucrose 2.3 ± 0.5 vs. placebo 1.6 ± 0.5, *p* = 0.6), heart rate variability (mean ± SD: sucrose 33 ± 6 vs. placebo 24 ± 5 bpm, *p* = 0.44), and crying time (mean ± SD: sucrose 63 ± 3 vs. placebo 49 ± 5 sec, *p* = 0.17) for both groups [30]. Similarly, a randomized trial with 66 preterm infants > 28 weeks gestation administered expressed breast milk or oral sucrose for pain management during venipuncture [31]. Overall, similar pain scores with 7 (range 4–9) with expressed breast milk and 6 (range 4–8) with sucrose were observed [31]. These studies suggest that oral sucrose might not be effective in all infants and that expressed breast milk has similar analgesic effects. Furthermore, the analgesia effect of sucrose might be ineffective in infants’ experience opioid withdrawal [45]. However, a recent study reported similar pain scores for opioid exposed and non-exposed infants suggesting that oral sucrose might be effective in both exposed and non-exposed infants [46].

There may, however, be negative effects of oral sucrose. Asmerom et al. randomized 131 premature infants undergoing a heel lance procedure to either control, placebo with NNS, or sucrose [47]. Although, a 22% decrease in median pain scores was observed with sucrose compared to control, increased markers of oxidative stress and increased use of adenosine triphosphate could indicate cellular injury in infants receiving sucrose. Furthermore, infants receiving sucrose had a significant increase in the heart rate from (mean ± SD): 155 ± 14 to 171 ± 155 bpm compared to infants the control (154 ± 13 to 155 ± 14 bpm) and placebo groups (156 ± 14 to 165 ± 15 bpm, *p* < 0.001) [47].

However, there might be also some positive long-term effects of sucrose on spatial learning and memory [48]. Rat models showed that chronic pain impaired short-term memory, but sucrose prevented such impairment and increased endorphin levels [48]. Sucrose also prevented a decrease levels of brain derived neurotropic factor, which occurs during chronic pain [48]. This conflicting evidence suggests that further studies are needed to examine long-term effects (e.g., long-term neurodevelopmental outcomes or obesity) of oral sucrose.

## 4. Discussion

Non-pharmacological techniques have the potential to provide pain relief for preterm and term infants. Most studies included in this review demonstrated an improvement in behavioral pain responses including facial expressions, duration of crying or latency to first cry, and physiological parameters (e.g., heart rate, oxygen saturation). This indicates that non-pharmacological techniques are beneficial and were successful at reducing pain. However, this finding was not reproducible in sucrose vs. placebo, breastmilk vs. sucrose, and breast feeding vs. musical therapy studies [20,30,31]. These contradictory results raise questions about the potential mechanism of these interventions. Further research is needed to determine the best non-pharmacological intervention, duration of the intervention, and dose response for optimal pain relief in newborn infants.

While sucrose is now considered the gold standard in non-pharmacological pain relief, the current evidence remains contradictory. Although, several studies identified a clear benefit of sucrose compared to other techniques (e.g., music, massage or NNS) [16,20,26,29], other studies reported lower pain score with alternative techniques including magnetic acupuncture or SSC [11,27]. The mechanism of sucrose has also been controversial. Many believe that sucrose decreases pain through opioid mechanisms but methadone-exposed newborn infants do not appear to be susceptible to the effects of sucrose, probably because of opioid-receptor blockade by methadone [45]. However, Marceau et al. reported that opioid exposed neonates had decreased pain responses with the use of sucrose which suggests additional other mechanisms for sucrose’s analgesic effects [46]. There is also conflicting evidence regarding any long-term effects of sucrose. Rat models reported that sucrose increased endorphins and brain-derived neurotrophic factor, which up-regulates neurogenesis and restores memory functions [48,49,50]. Interestingly, an increase in oxidative stress markers, which might lead to cellular damage was also reported after sucrose administration [47]. This raises questions as to whether sucrose can be should be the gold standard for non-pharmacological pain relief and studies examining long-term effects are needed.

The main limitation of the studies included in this review was the differences in the way pain was assessed. In preterm infants, heart rate or oxygen saturation variations may also have been due to physiological immaturity, entirely unrelated to the painful procedure or to the intervention. There is no standardized approach to the measurement of pain and each study used different scores e.g., Premature Infant Pain Profile score, Neonatal Infant Pain Scale score, Douleur Aiguë du Nouveau-né scale (a French scale of neonatal pain) [24], Face, Legs, Activity, Cry and Consolability Pain Scale [30], which makes it impossible to compare studies in a meta-analysis.

There are also limitations to the effectiveness of the non-pharmacological methods depending on many factors. Studies suggest that gestational age might influence the response to non-pharmacological treatments. Studies comparing sucrose with breast milk reported similar pain scores in infants >28 weeks’ gestation [23], while infants <28 weeks’ gestation had significantly lower pain scores after oral sucrose but not after breast milk [31]. Skin-to-skin care, swaddling and facilitated tucking is limited to times when the infant’s position is not crucial and is not suitable for emergency procedures or for procedures dependent on position (e.g., lumbar puncture). Breastfeeding also may not always be available (e.g., in NICU or mother not present). If the infant is very ill, a lot of the interventions may not be possible.

One of the major advantages to using these non-pharmacological methods is their high safety profile. Most importantly, all these interventions have a very favorable benefit to risk ratio, even if the benefits are modest—the risk is extremely low. Supervision and support from staff helps with safety concerns for SSC, swaddling, massage therapy, facilitated tucking and BF such as improper technique, holding or positioning [51]. When using laser acupuncture, the infant and acupuncturists must wear protective ear-gear as well as cover any reflective surface to prevent the laser from hitting one’s eye. Yates et al. assessed the safety of non-invasive electrical stimulation at acupuncture points (NESAP) by observing skin reactions, vital signs and PIPP scores [52]. There were no significant changes found in these measures and no adverse events occurred, which concludes that NESAP is safe [52]. Some of the main challenges in implementation is reluctance from staff or parents, due to disbelief or the invasiveness of the procedure (e.g., use of needle acupuncture in infants).

The major benefit of non-pharmacological treatments includes (i) ease of use, (ii) apparent safety, (iii) feasibility, and (iv) ease of learning, which would allow universal implementation of any of these interventions. However, acupuncture using needle or laser would require training and experience about the specific acupuncture points, and lasers might not be readily available. The long-term effects of any non-pharmacological intervention have not been studied and is a major knowledge gap, that needs to be addressed.

## 5. Conclusions

Newborn infants in an NICU undergo many painful but necessary procedures during hospitalizations. The implications of the pain associated with these procedures and the types of pain relief given to the infants have considerable implication for both short- and long-term outcomes. The evidence for non-pharmacological analgesia is sparse and needs further study. While most appear to be safe and relatively effective, their effects on the long-term outcomes of the infants is unknown, especially when coupled with pharmacological analgesia. 

## Figures and Tables

**Table 1:** Non-pharmacological treatments for pain relief.

**Environmental Control**
Skin-to-skin care Swaddling Facilitated tucking Therapeutic touch/massage Musical therapy
**Feeding Methods**
Non-nutritive sucking Breastfeeding
**Other Interventions**
Acupuncture Sucrose/glucose solutions

**Table 2:** Randomized controlled trials for non-pharmacological methods of pain relief in infants.

First Author, Year	Population	Intervention	Other Intervention	Primary Outcome		*p*-Value
				Intervention	Control	
**Shabani, 2016 [9]**	Preterm (*n* = 20)	MT	N/A	Facial pain expressions M (SD): MT: 0.4 (0.1)	Control: 2.1 (0.4)	0.001
**Seo, 2016 [10]**	Term (*n* = 56)	SSC	N/A	PIPP M ± SD: SSC: 4.1 ± 2.3	Control: 6.3 ± 3.5	0.01
**Freire, 2008 [11]**	Preterm (*n* = 95)	SSC	Glucose	HR M ± SD: SSC: 5.1 ± 3.9 bpm Glucose: 9.9 ± 6.1 bpm	Control: 10.8 ± 6.5 bpm	SSC vs. glucose: 0.0001 SSC vs. control: 0.0001
**Olsson, 2016 [12]**	Preterm (*n* = 10)	SSC	N/A	PIPP M: 5.7	Control: 5.0	>0.05 (NS)
**Efendi, 2018 [13]**	Preterm (*n* = 30)	Pacifier and swaddling	N/A	Increase in pain score: Swaddle: 5.9 ± 2.2 to 6.1 ± 2.0	Control: 5.4 ± 1.8 to 7.7 ± 2.7	Swaddling: NS Control: 0.003
**Erkut, 2017 [14]**	Term (*n* = 74)	Swaddle	N/A	NIPS M ± SD: Swaddle: 1.6 ± 0.8	Control: 3.3 ± 1.5	0.01
**Ho, 2016 [15]**	Preterm (*n* = 54)	Swaddle	N/A	PIPP M ± SD: Swaddle: 7.0 ± 2.7	Control: 14.7 ± 2.9	<0.001
**Axelin, 2006 [16]**	Preterm (*n* = 20)	FT	N/A	NIPS Median (IQR): FT: 3 (2–6)	Control: 5 (2–7)	<0.001
**Arikan, 2008 [17]**	Preterm/term (*n* = 175)	Massage	Sucrose herbal tea, hydrolyzed formula	Crying time after procedure M ± SD: Massage: 4.4 ± 1.8 s Sucrose: 3.9 ± 1.5 s Tea: 3.2 ± 1.2 s Formula: 2.7 ± 1.1 s	Control: 5.3 ± 1.76 s	Comparing before and after procedure: *p* < 0.001 for all but control (*p* > 0.05)
**Chik, 2017 [18]**	Preterm/term (*n* = 80)	Massage	N/A	PIPP M (SD): Massage: 6 (3.3)	Control: 12 (4.3)	<0.001
**Jain, 2006 [19]**	Preterm (*n* = 23)	Massage	N/A	NIPS M (SD): Massage: 1.5 (0.9)	Control: 3.5 (1.6)	<0.001
**Zhu, 2015 [20]**	Term (*n* = 250)	MT	BF	NIPS M (SD): MT: not significant MT + BF: not significant BF: 3.1 (1.9)	Control: 6.4 (0.2)	BF vs. control: <0.001
**Shah, 2017 [21]**	Preterm/term (*n* = 35)	MT	Sucrose	PIPP median (IQR): MT: 6 (3–11) MT + sucrose: 3 (0–4)	Sucrose: 5 (3–10)	MT vs. sucrose: >0.05 MT + sucrose vs. sucrose: <0.001 MT + sucrose vs. MT: <0.001
**Zurita-Cruz, 2017 [22]**	Term (*n* = 144)	BF	MS	Crying time Median (IQR): BF: 19 (0–136) MS: 41.5 (0–184)	Control: 41 (0–161)	BF vs. control: 0.007 Control vs. MS: >0.05
**Erkul, 2017 [23]**	Term (*n* = 100)	BF	N/A	NIPS M ± SD: BF: 1.9 ± 2.2	Control: 6.8 ± 0.7	<0.05
**Simonse, 2012 [24]**		BF	Bottle fed, sucrose	PIPP M (95%CI): BF: 7.0 (5.3–8.7) Bottle fed: 5.4 (3.7–7.1)	Sucrose: 5.3 (3.6–6.9)	BF vs. bottle fed: >0.05 BF vs. sucrose: >0.05
**Baudesson, 2017 [25]**	Preterm (*n* = 33)	MO	N/A	PIPP M (SD): MO: 7.3 (3.0)	Control: 10 (3.5)	0.03
**Mitchell, 2016 [26]**	Term (*n* = 162)	NESAP	Sucrose	PIPP M ± SD: NESAP: 5.0 ± 4.0 Sucrose: 4.0 ± 1.8 NESAP + sucrose: 3.6 ± 1.2	Control: 4.9 ± 4.0	<0.01
**Chen, 2017 [27]**	Preterm/term (*n* = 30)	MA	N/A	PIPP M ± SD: MA: 5.9 ± 3.7	Control: 8.3 ± 4.7	0.04
**Abbasoglu, 2015 [28]**	Preterm (*n* = 32)	Acupressure	N/A	PIPP M ± SD: Acupressure: 9.1 ± 2.0	Control: 9.6 ± 1.7	0.5
**Lima, 2017 [29]**	Term (*n* = 78)	NNS	Glucose	NIPS M ± SD: NNS: 33.9 ± 17.6	Glucose: 10.9 ± 11.3	<0.001
**Gouin, 2018 [30]**	Term (*n* = 245)	Sucrose	N/A	NIPS M ± SD: Sucrose: 2.3 ± 0.5	Control: 1.6 ± 0.5	0.6
**Collados-Gómez, 2018 [31]**	Preterm (*n* = 66)	Sucrose	EBM	PIPP Median (IQR): Sucrose: 6 (4–8)	EBM: 7 (4–9)	0.28

M: mean, SD: standard deviation, IQR: interquartile range, NS: not significant, HR: heart rate, SSC: skin-to-skin care, FT: facilitated tuck, MT: musical therapy, BF: breast feeding, MO: milk odor, NS: non-significant, NNS: non-nutritive sucking, NESAP: non-invasive electrical stimulation at acupuncture points, N/A: not/applicable, MS: milk substitute, EBM: expressed breastmilk, PIPP: Premature Infant Pain Profile, NIPS: Neonatal Infant Pain Scale, bpm: beats per minute.

## Appendix A

Search of PubMed—last performed 4 June 2018	
#1	infant (*n* = 1,119,189)
#2	infants (*n* = 1,159,867)
#3	preterm (*n* = 62,246)
#4	premature (*n* = 170,785)
#5	pain (*n* = 733,273)
#6	relief (*n* = 84,761)
#6	acupuncture (*n* = 28,139)
#7	skin-to-skin care (*n* = 47,999)
#8	non-nutritive sucking (*n* = 367)
#9	sucrose (*n* = 74,579)
#10	massage (*n* = 13,982)
#11	music (*n* = 22,562)
#12	breastfeeding (*n* = 49,518)
#13	non-pharmacological (*n* = 6513)
Search of Google Scholar—last performed 4 June 2018	
#1	infant (*n* = 3,220,000)
#2	infants (*n* = 2,080,000)
#3	preterm (*n* = 1,010,000)
#4	premature (*n* = 2,840,000)
#5	pain (*n* = 3,770,000)
#6	relief (*n* = 2,390,000)
#6	acupuncture (*n* = 592,000)
#7	skin-to-skin care (*n* = 3,820,000)
#8	non-nutritive sucking (*n* = 21,900)
#9	sucrose (*n* = 2,640,000)
#10	massage (*n* = 773,000)
#11	music (*n* = 3,770,000)
#12	breastfeeding (*n* = 649,000)
#13	non-pharmacological (*n* = 2,540,000)
